# Editorial: A Good Sleep: The Role of Factors in Psychosocial Health

**DOI:** 10.3389/fnins.2020.00520

**Published:** 2020-05-26

**Authors:** Amir H. Pakpour, Mark D. Griffiths, Maurice M. Ohayon, Anders Broström, Chung-Ying Lin

**Affiliations:** ^1^Social Determinants of Health Research Center, Research Institute for Prevention of Non-communicable Diseases, Qazvin University of Medical Sciences, Qazvin, Iran; ^2^Department of Nursing, School of Health and Welfare, Jönköping University, Jönköping, Sweden; ^3^International Gaming Research Unit, Psychology Department, Nottingham Trent University, Nottingham, United Kingdom; ^4^Stanford Sleep Epidemiology Research Center, School of Medicine, Stanford University, Stanford, CA, United States; ^5^Department of Clinical Neurophysiology, Linköping University Hospital, Linköping, Sweden; ^6^Department of Rehabilitation Sciences, Hong Kong Polytechnic University, Hung Hom, Hong Kong

**Keywords:** sleep, psychosocial, mental health, child, adolescent, adults

A good night's sleep is vital for individuals of all ages to have effective cognitive and emotional processing (Kopasz et al., 2010; Yaffe et al., 2014; Garbarino et al., 2016; Tarokh et al., 2016). Furthermore, prior evidence shows that sleep is associated to physical and mental health, and to overall quality of life (Gradisar et al., 2008; Shochat et al., 2014; Garbarino et al., 2016), and therefore a good sleep is of great importance (Lin C.-Y. et al., 2018; Lin P.-H. et al., 2018). Unfortunately, it is not always easy for many people to achieve good sleep (Strong et al., 2018), especially in modern society that has rapid growth in technology. Indeed, a recent systematic review and meta-analysis found that internet addiction is highly associated with sleep disturbance (Alimoradi et al., 2019). Similarly, recent research shows the association between problematic social media use and poor sleep, which indicates a contemporary public health problem concerning sleep (Wong et al., 2020). In short, there is a need to investigate how different psychosocial factors are related to sleep in different stages of life.

Therefore, this special issue focuses on a variety of psychosocial factors associated with sleep in different age groups and contexts and comprises a systematic review and meta-analysis together with eight empirical papers. The systematic review and meta-analysis investigated by Magnavita et al. screened 749 studies, of which 34 were reviewed and seven were included in meta-analysis. They concluded that sleep problems could be increased by workplace violence (OR = 2.55; 95% CI = 1.77–3.66). In addition to the systematic review and meta-analysis, the other eight studies included in the special issue demonstrate the variety of different psychosocial factors that contribute to sleep across different populations. More specifically, four studies comprised Taiwanese populations, including female college students (Lin et al.), adolescents (Ho et al.; Hsieh et al.), and children (Lin), one study comprised Hong Kong children (Chien et al.), one study comprised the Polish general population (Herzog-Krzywoszanska and Krzywoszanski), and two studies comprised Swedish adolescents (Hedin et al.; Hena and Garmy).

Lin et al. recruited 503 female college students and found that students with a moderate to severe level of internet addiction had significantly poorer sleep quality than did those with mild or normal levels of internet addiction. Furthermore, those with mild levels of internet addiction had significantly poorer sleep quality than those not addicted to the internet. Logistic regression analysis further demonstrated the association between internet addiction and sleep quality (odds ratio = 1.05 95% CI = 1.03–1.06, *p* < 0.01). These findings echo the findings of the aforementioned systematic review and meta-analysis (Alimoradi et al., 2019).

Ho et al. utilized a longitudinal secondary dataset (i.e., Taiwan Youth Project; Yi et al., 2009) and a marginal structural model with stabilized inverse probability-of-treatment weights to investigate the association between unhealthy sleep practice and substance use. Their findings indicated some degree of causality showing that unhealthy sleep practice leads to adulthood substance use. A similar association between sleep and alcohol abuse was found in another study comprising Taiwanese adolescents (Hsieh et al.) Hsieh et al. used data from the 2009 Project for the Health of Children and Adolescents in Southern Taiwan (Yen et al., 2010) and found that insomnia may result from alcohol abuse, suicidality, depression, and anxiety among 6,445 high school students.

Lin collected information from 320 Taiwanese child-parent dyads in a community-centered elementary school using a 12-week longitudinal design. She used an instrument with objective measures (i.e., a Xiaomi Mi Band 2 pedometer; Xie et al., 2018) to assess children's sleep over 12 consecutive weeks (i.e., the children were requested to wear the pedometer on their wrists during the 12 weeks). Moreover, parents of the children completed the Kid-KINDL (Lin et al., 2017; Lin, 2018) to assess the children's quality of life before they wore the pedometer. Her results found that better quality of life may lead to better sleep.

Chien et al. found in a study comprising 391 Hong Kong children that homework involvement was positively related to children's weekday sleep duration, and that frequency of television watching was negatively related to their weekday sleep duration. Moreover, overall participation in school activities was positively related to children's weekend sleep duration. However, given that Chien et al. used cross-sectional design, the causality was undetermined.

Herzog-Krzywoszanska and Krzywoszanski used two Polish samples to investigate factors related to bedtime procrastination. Their first sample comprised university students (*n* = 431) and their data were utilized to validate a Polish version scale of the Bedtime Procrastination Scale (BPS; Kroese et al., 2016). After confirming the good psychometric properties of the BPS, Herzog-Krzywoszanska and Krzywoszanski found that studying or working needs may delay bedtime in their second sample of general population (*n* = 335). However, similar to the study of Chien et al., the study was cross-sectional and cannot provide strong evidence of causal relationships.

Hena and Garmy used a cross-sectional study on Swedish adolescents (*n* = 1,518) and found that social jetlag (defined as the difference between bedtime and wake-up time on school days compared to holidays larger than 2 h; Wittmann et al., 2006) was significantly associated with increased screen time. This echoes Lin et al. 's findings of internet addiction's association with poor sleep. In the final paper, Hedin et al. conducted a qualitative study exploring the facilitators and barriers for a good night's sleep among adolescents (*n* = 45). Their findings indicated that adolescents understood and appreciated commonly recommended strategies for improving sleep. However, it was hard to balance their sleep and other activities. Consequently, they concluded that assisting adolescents to overcome the dilemma of finding a balance between sleep and other activities is crucial when designing sleep-promotion interventions.

## Author Contributions

C-YL wrote the first draft. AP, MG, MO, and AB provided critical comments and editorial suggestions for revisions. All the authors agreed on the submitted version.

## Conflict of Interest

The authors declare that the research was conducted in the absence of any commercial or financial relationships that could be construed as a potential conflict of interest. The handling Editor declared a shared affiliation with one of the authors, MO.
